# Changes in eating behavior traits and diet in older prediabetic men during a 3-year lifestyle intervention

**DOI:** 10.1007/s00394-025-03876-7

**Published:** 2026-03-05

**Authors:** Noora Koivu, Maria Lankinen, Ursula Schwab

**Affiliations:** 1 Institute of Public Health and Clinical Nutrition, University of Eastern Finland, P.O. Box 1627, FI-70211 Kuopio, Finland; 2 Department of Medicine, Endocrinology and Clinical Nutrition, Kuopio University Hospital, Wellbeing Services County of North Savo, Kuopio, Finland

**Keywords:** Eating behavior, Cognitive restraint, Emotional eating, Uncontrolled eating, Diet, Lifestyle intervention

## Abstract

**Purpose:**

Eating behavior traits are linked to diet, but how changes in these traits are associated with dietary changes, particularly in men, remains unclear. Understanding these associations may help develop personalized prevention and treatment strategies for non-communicable diseases such as type 2 diabetes. This study aimed to examine how eating behavior traits and food intake change in older Finnish men with prediabetes during a lifestyle intervention, and to explore the associations between these changes.

**Methods:**

The Three-Factor Eating Questionnaire-R18 was used to assess three eating behavior traits (cognitive restraint, uncontrolled eating and emotional eating), and repeated 4-day food records were used to evaluate food intake in 368 prediabetic men aged 50–75 during a 3-year lifestyle intervention.

**Results:**

During the intervention, cognitive restraint increased while uncontrolled eating and emotional eating decreased (FDR-*p* < 0.001 for all). The intake of fiber-rich foods increased and the intake of foods rich in saturated fat decreased. A decrease in uncontrolled eating associated with decreases in the intake of sweet (FDR-*p* = 0.02) and savory pastries (FDR-*p* = 0.01) and chocolate (FDR-*p* = 0.02). A decrease in emotional eating was associated with a higher intake of vegetables, fruit and berries (FDR-*p* = 0.01).

**Conclusions:**

A decrease in uncontrolled and emotional eating plays a role in increased intake of vegetables, fruit and berries and decreased intake of energy-dense foods. The findings are essential to consider when developing effective prevention and treatment strategies for type 2 diabetes.

**Supplementary Information:**

The online version contains supplementary material available at 10.1007/s00394-025-03876-7.

## Introduction

Globally, 541 million adults are at high risk for type 2 diabetes (T2D) [1], which is among the top ten leading causes of death [2]. Aging increases the risk of T2D [3, 4]. T2D has been more common among older (aged 45–74) men than women in various populations [5–8] and ethnic groups within the UK Biobank study [9]. Healthy lifestyles, such as high-quality diet, physical activity, and weight management are crucial for preventing and treating T2D [10]. Lower-quality diet has been independently associated with a higher risk of T2D [11, 12], and it also plays a key role in weight management [13]. Most adults in high-income Western countries fail to adhere to national nutrition recommendations, particularly regarding the quality of dietary fat and the intake of fiber, vegetables and fruit [14–16]. In Finland, men are even further away from the recommendations than women. Thus, new means with long-term impact are needed to improve dietary quality and prevent T2D and other non-communicable diseases.

Our food choices are affected by more than just conscious deliberation [17]. Eating behavior is a complex phenomenon that encompasses all eating-related actions, decisions, and habits [18]. Eating behavior traits, such as cognitive restraint (CR, i.e. conscious restriction of intake of certain foods or energy, to control body weight), emotional eating (EE, i.e. regulating feelings by eating) and uncontrolled eating (UE, i.e. overeating as a result of loss of control) [19], describe individual eating patterns [20]. Since eating behavior traits have been associated with diet quality in cross-sectional studies, they may influence both quantity and quality of diet: CR has been linked to higher intake of health-beneficial foods such as fruit, vegetables, and fish in various populations [21–26], while UE has been associated with higher consumption of [27, 28] and preference for [29] energy-dense foods, and EE with greater intake of sweet energy-dense foods [22, 23]. However, the participants in these studies have mainly been women. In few existing longitudinal studies, an increase in CR has been associated with dietary improvements, but it remains unclear how changes in UE and EE affect diet quality [30–32]. Therefore, these associations should be studied further, particularly in men. In the limited number of interventions that have examined eating behavior traits among prediabetic individuals, dietary restraint increased [33, 34] and EE decreased over six to twelve months [33]. During these interventions, diet quality improved, i.e., the quality of dietary fat improved [35] and the intake of fruit and vegetables increased [35, 36]. However, the longitudinal associations between eating behavior traits and diet have not been analyzed. Understanding these associations could provide valuable insights for the development of more personalized and effective prevention and treatment strategies for non-communicable diseases such as T2D.

Aging men are underrepresented in eating behavior research, and findings from studies on women may not be directly generalized to men due to potential sex differences in eating behaviors: Women tend to report higher levels of CR, UE and EE compared to men [37–39]. The relationships between eating behavior traits and diet quality may also differ by sex: UE has been associated with higher energy intake only in women [22], while CR has been associated with higher cheese intake and lower alcohol intake exclusively in men [21, 22]. EE has been related to the intake of both savory and sweet fatty foods [22, 40, 41] but potentially stronger to sweet fatty foods in women [42].

T2D-GENE study was a 3-year lifestyle intervention that focused on health-promoting diet and exercise to prevent T2D in aging prediabetic men [43–45]. The intervention provides valuable data for investigating changes in eating behavior traits and diet among older men. Few studies have examined the eating behaviors of aging men, and even fewer, if any, have explored their longitudinal associations with diet quality. Thus, to better understand the role of eating behavior traits in dietary changes we aimed to study: (1) how eating behavior traits and food consumption change during a 3-year lifestyle intervention among aging prediabetic men and (2) the associations between the changes in eating behavior traits and in food consumption during the intervention. We hypothesize that: (1) CR scores will increase during the intervention, while UE and EE scores will decrease, alongside improvements in dietary intake, and (2) changes in CR scores will be directly associated with changes in the consumption of health-promoting foods, whereas changes in UE and EE scores will be directly associated with changes in the consumption of energy-dense foods.

## Methods

### Study design and participants

This is a longitudinal analysis of the T2D-GENE lifestyle intervention [43–45]. The aim of the T2D-GENE intervention study was to investigate whether a low or high genetic risk affects the development of T2D in a group-based lifestyle intervention [45]. Overall, the intervention was successful, reducing the risk of T2D by 52% [45].

**Fig. 1:**
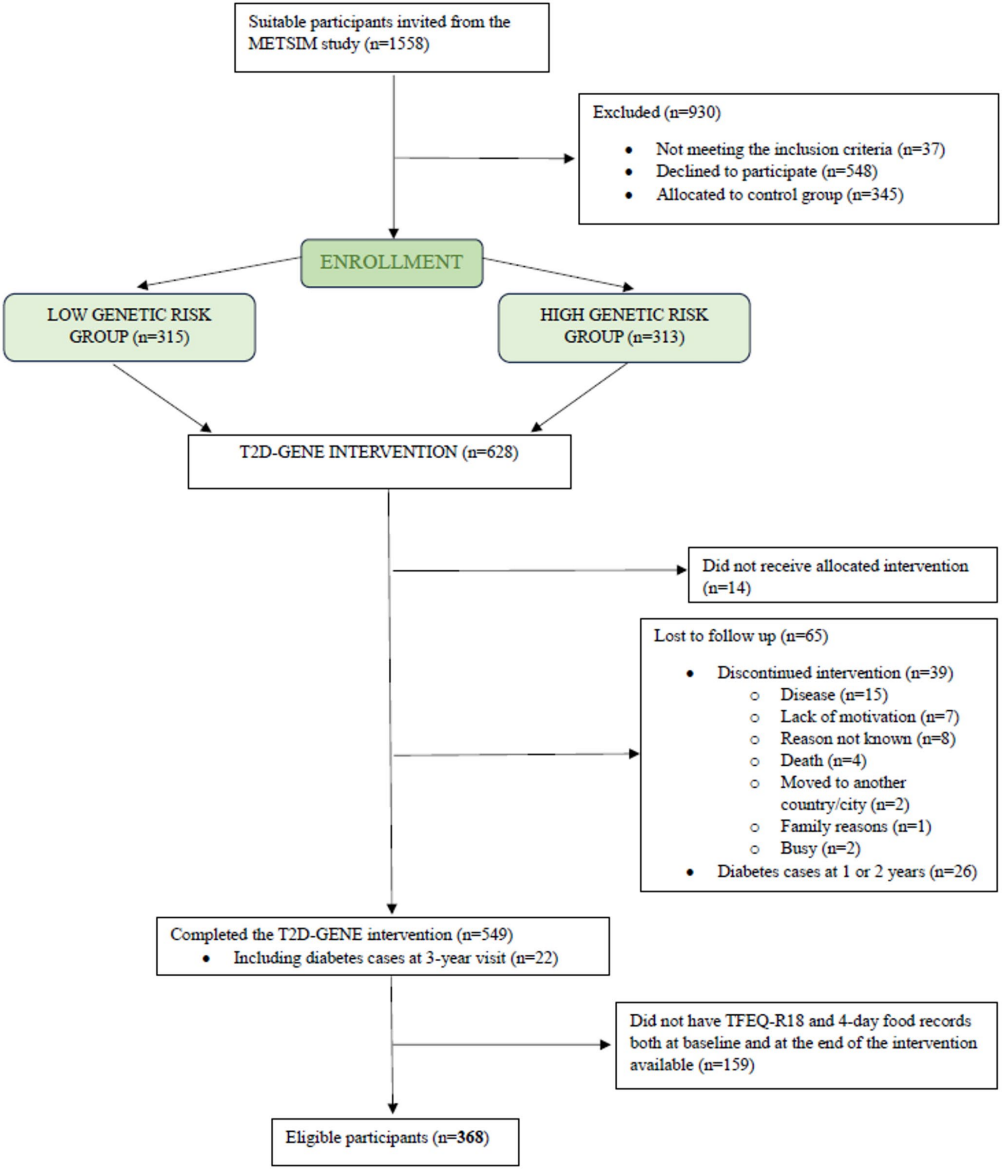
Flowchart. TFEQ-R18, the three-factor eating questionnaire revised 18-item

Participants in the T2D-GENE intervention were recruited from the Metabolic Syndrome in Men-study (METSIM) cohort (Fig. 1) [46]. The participants were randomly selected volunteers from the population register of the Kuopio town, Eastern Finland. The inclusion criteria for the T2D GENE study were: (1) impaired fasting glucose (IFG) (fasting plasma glucose ≥ 5.6 mmol/l) with impaired glucose tolerance (IGT) (2-hour glucose 7.8–11.0 mmol/l) or without IGT (2-hour glucose < 7.8 mmol/l), and HbA1c < 48 mmol/mol (< 6.5%), (2) age 50–75 years, (3) overweight or obesity (Body mass index (BMI) > 25 kg/m^2^) and (4) low or high genetic risk for T2D. The genetic risk score was calculated based on the number of risk alleles of 76 genes that are known to increase risk for T2D [47]. The participants were distributed into three tertiles according to their risk status: high, intermediate and low genetic risk of T2D. People with intermediate genetic risk were excluded from the study because the aim of the T2D-GENE study was to compare the effects of low and high genetic risk groups [45]. Chronic diseases or conditions, such as chronic liver, thyroid and kidney diseases, that might hamper the participation in the study were the exclusion criteria.

Altogether, 614 participants were recruited for the T2D-GENE intervention and received the allocated intervention (Fig. 1). The control group of 345 participants did not complete the food records or eating behavior questionnaires and therefore were excluded from the analyses. The participants of the intervention were assigned to either the highest tertile (high genetic risk group *n* = 303) or the lowest tertile (low genetic risk group *n* = 311) based on their genetic risk score. The participants, laboratory nurses and clinical nutritionists were blinded to these assignments. In our study the genetic risk groups were not considered. Of the 614 participants, 368 men had food records and The Three-Factor Eating Questionnaire Revised 18-item (TFEQ-R18) data available at baseline and at three years and none had been diagnosed with T2D before the 3-year visit; these participants were included in our study. Baseline characteristics of the 368 participants are presented in Table 1. The 246 dropouts were similar to those included in the analysis, except that the dropouts were younger (64.4 years ± 5.7) (FDR-*p* = 0.03) and had higher blood HbA1c (37.9 mmol/mol ± 3.0; 5.6%; 0.3) (FDR-*p* = 0.03).

**Table 1:** Baseline characteristics in the T2D-GENE intervention (*n* = 368), Mean *±* SD or n (%)

Age (years)	65.7 ± 5.9
BMI (kg/m^2^)	28.5 ± 3.0
Fasting plasma glucose (mmol/l)	6.0 ± 0.3
2-hour plasma glucose (mmol/l)	6.2 ± 1.5
Blood HbA1c (mmol/mol (%))	37.2 ± 3.2 (5.6 ± 0.3)
Education ^a^	
Primary school	67 (18.9)
Secondary school	202 (56.9)
Higher level	71 (20)
Other	15 (4.2)
Marital status ^a^	
Married/registered partnership/cohabiting	309 (87)
Single/divorced/widow/living apart	46 (13)
Responsibility of food management ^b^	
With a partner	286 (80.3)
Alone	44 (12.4)
Not at all	26 (7.3)

BMI, Body mass index; HbA1c, Hemoglobin A1c

^a^ *n* = 355

^b^ *n* = 356

### T2D-GENE intervention

**Fig. 2:**
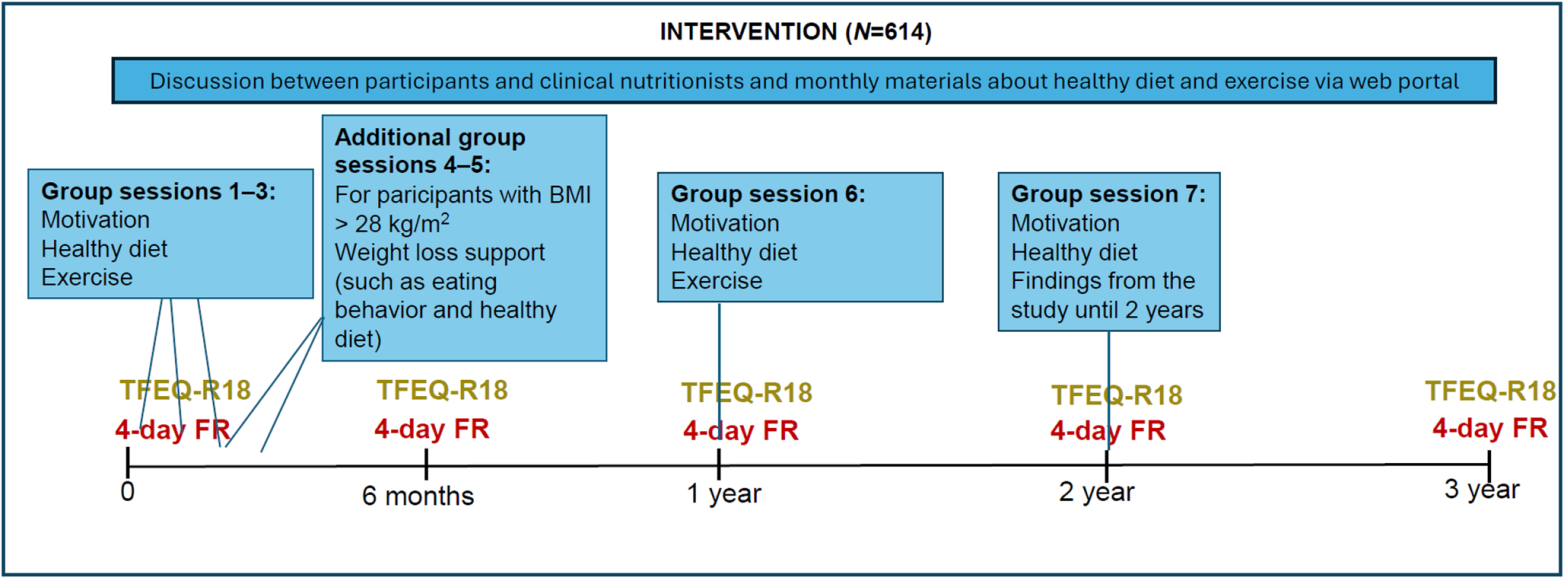
Study design. TFEQ-R18, the three-factor eating questionnaire revised 18-item; FR; Food Record

The T2D-GENE intervention was conducted in 2016–2021 [43–45]. The 3-year intervention has been described in detail elsewhere [43, 44]. In brief, the intervention included 5–7 group sessions for motivation and to increase knowledge about health-promoting lifestyle (Fig. 2). The precise timing, topics and participation rates of the sessions are outlined in previous publications [43, 44]. The group sessions, led by clinical nutritionists, focused on healthy diet, physical activity and the importance of lifestyle changes to reduce the risk of T2D. The participants needing support for weight loss (BMI > 28 kg/m^2^) had two additional meetings that addressed weight loss and the prevention of weight gain. These meetings included topics related to eating behaviors, such as mindful eating, flexible restraint of eating and risk factors for overeating, in addition to a healthy diet. Along with the group sessions, the participants had access to a web portal providing monthly material about health promoting diet and physical activity. Laboratory visits were conducted at the baseline and at one, two, and three years.

### Eating behaviors

The self-reported TFEQ-R18 [19] (Supplementary Material 1) was used to assess the intensity of three eating behavior traits: CR, UE, and EE. These traits were assessed five times during the intervention: at baseline, 0.5, 1, 2 and 3 years (Fig. 2). For each eating behavior trait, the score ranges from 0 to 100, with higher scores indicating greater intensity of the trait. TFEQ-R18 has been validated in individuals with and without obesity [19, 22, 48]. In this study, the Finnish version of the TFEQ-R18 was used, which has been translated and back-translated by the Finnish Association for the Study of Obesity from the original version. The Finnish version has demonstrated good structural validity among young Finnish females with diverse BMIs [49].

Although the T2D-GENE intervention did not focus on changing eating behavior traits, the two additional group meetings for the participants with a BMI over 28 kg/m² covered topics related to diet quality, food energy density, and strategies for managing eating behaviors [43, 44].

Previously, it was proposed that a modified 15-item version could be used instead of R18, for aging men with increased risk for T2D, as this version was found to better fit the needs of this population [50]. The 15-item version excluded two items from the CR factor and one item from the UE factor. The analyses were performed with both versions and the results were very similar. Therefore, we decided to report only the results from the R18 version for better comparability with previous studies.

### Diet

Dietary guidance was based on the Nordic and the Finnish nutrition recommendations [51]. The dietary intake was assessed through self-reported 4-day food records with four consecutive days, including one weekend day. These food records were completed five times throughout the intervention, i.e. at baseline, 0.5, 1, 2 and 3 years (Fig. 2). The participants were instructed to report all foods and beverages consumed, including specific quantities and preparation methods. To avoid loss of accuracy, the participants were advised to weigh their food, estimate the weights of foods using an electronic picture book of portion sizes, or employ household measures. The food records were reviewed face-to-face by clinical nutritionists to enhance accuracy. Dietary intake was calculated using AivoDiet software version 2.2.0.0 (Mashie FoodTech Solutions Finland Oy, Turku, Finland). In addition to energy and nutrients, all the foods and ingredients were listed from the food records. Based on the calculations, the participants received written feedback and if needed, individual instructions for dietary improvements.

Foods from the 4-day food records were analyzed both separately and grouped into 11 food groups to assess overall dietary patterns (Supplementary Material 2). These groups were based on central nutritional principles aimed at preventing and treating T2D, such as the preference of mono- and polyunsaturated fats over saturated or trans fat, increased dietary fiber intake, and, for people with overweight or obesity, restriction of energy intake [52]. Food consumption is reported as grams per day. We analyzed 11 food groups, the individual foods within these groups, as well as individual foods not included in any group. This allowed us to examine the effects of both grouped and separate food items on overall dietary composition and other variables of interest.

### Statistical methods

Baseline characteristics are presented as means ± SDs and percentages. Differences between the participants and the dropouts were assessed with the Mann-Whitney U test and Chi-Square test.

Eating behavior traits and food intake variables are reported as means ± 95% CIs at baseline and at three years. None of these variables were normally distributed. Differences between baseline and three-year values (delta values) were calculated for eating behavior traits, food categories and from the individual food items within each category (Supplementary Material 2). Wilcoxon signed-rank test was used to evaluate the significance of changes in eating behavior traits and food intake.

Correlations between delta values of eating behavior traits and the dietary factors (food categories and foods within the categories, expressed in grams per day) were assessed using Pearson’s correlations, as the delta values were normally distributed. The delta values of dietary factors that showed significant correlations with the delta values of eating behavior traits were selected for further analysis using linear regression models to examine how changes in eating behavior traits predict changes in food intake. In these models, the changes in eating behavior traits were independent variables and the change in the intake of a food variable was the dependent variable. Baseline BMI was included as a covariate due to its established association with both eating behavior traits and dietary intake. The main assumptions for linear regression were met: the residuals of all models were normally distributed and linear, there was no autocorrelation, and multicollinearity was absent (VIF values < 5).

Multiple comparison correction was performed using Benjamini–Hochberg False Discovery Rate correction to control for multiple testing. P-values were FDR-p corrected except for those related to the correlations (Supplementary Material 3), which were used as a screening tool to identify potential associations for further examination using linear regression models. The number of comparisons conducted was 8 for baseline characteristics, 19 for food variables, 3 for eating behavior traits, and 24 for linear regression models. FDR-*p* < 0.05 was considered statistically significant. All statistical analyses were performed using SPSS for Windows, version 27.0 (IBM Inc., Armonk, NY), except for FDR corrections for multiple comparisons, which were performed using R, version 4.3.1 (R Foundation for Statistical Computing, 2023).

## Results

### Changes in eating behavior traits and food intake

**Fig. 3:**
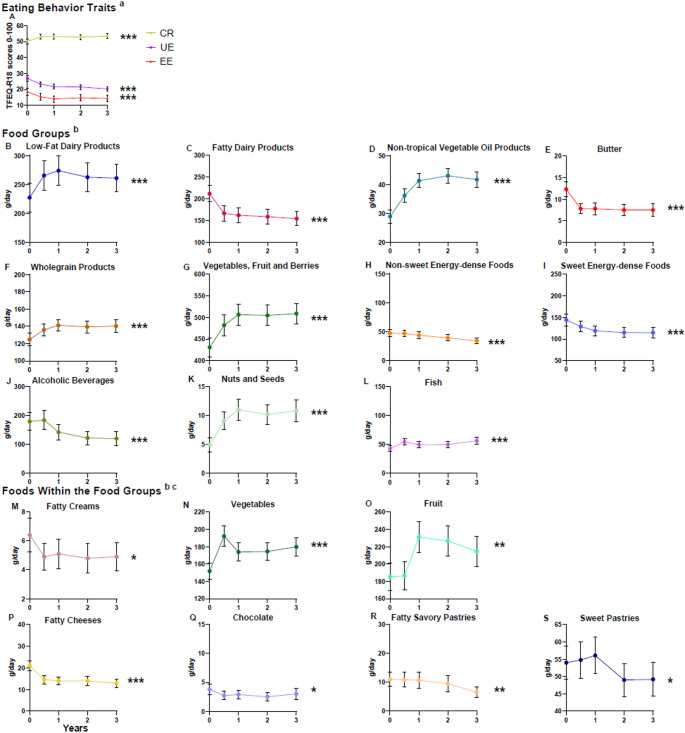
Changes in Eating Behavior Traits and Dietary Intake During the T2D-GENE Intervention Measured with Wilcoxon’s Signed Rank Test, Means and 95% confidence intervals (CIs) (*n* = 368). CR, Cognitive restraint; UE, Uncontrolled eating; EE, Emotional eating; TFEQ-R18, The Three-Factor Eating Questionnaire Revised 18-item, ^a^ n0 = 368, n0.5 = 323, n1 = 326, n2 = 349, n3 = 368. ^b^ n0 = 368, n0.5 = 362, n1 = 360, n2 = 363, n3 = 368, ^c^ Changes in the intake of foods that had significant associations with eating behavior traits. FDR-*p* < 0.05*, FDR-*p* < 0.01**, FDR-*p* < 0.001***.

Out of the three eating behavior traits, the participants exhibited the highest scores for CR (Fig. 3). Over the 3-year intervention, CR increased, while EE and UE decreased (FDR-*p* < 0.001 for all). There was no significant change in total energy intake during the intervention (FDR-*p* = 0.64). The intake of low-fat dairy products, vegetables, fruit, and berries, whole-grain products, vegetable oil products and fish increased significantly (FDR-*p* < 0.001 for all). Furthermore, the intake of fatty dairy products, butter and spreads containing butter, non-sweet energy-dense foods, sweet energy-dense foods, and alcoholic beverages decreased (FDR-*p* < 0.001 for all). Only those foods within the food groups that had significant associations with eating behavior traits (Table 2) are presented in Fig. 3. The intake of vegetables (FDR-*p* < 0.001) and fruit (FDR-*p* = 0.004) increased, and the intake of fatty creams (FDR-*p* = 0.04), fatty cheeses (FDR-*p* < 0.001), sweet pastries (FDR-*p* = 0.04), fatty savory pastries (FDR-*p* = 0.004), and chocolate (FDR-*p* = 0.01) significantly decreased. Supplementary Material 4 presents the magnitude of dietary changes (g/day).

### The associations between changes in eating behavior traits and changes in diet

Pearson’s correlation analysis was conducted to screen for potential associations between changes in eating behavior traits and changes in dietary intake. Due to the large number of food items from the food records, only the dietary variables that showed significant correlations and were relevant to the participants’ consumption patterns are presented in Supplementary Material 3. These variables were then selected for further analysis using linear regression. The change in CR was inversely correlated with the change in the intake of whole-grain products. A direct correlation was observed between the change in UE and the change in the intakes of sweet pastries, fatty savory pastries and chocolate. The change in EE correlated inversely with the change in the intakes of vegetables, fruit and berries as a food group as well as vegetables, and fruit separately, and fatty creams. The change in EE was directly correlated with fatty cheeses.

All significant associations identified in the linear regression analysis are presented in Table 2. The increase in CR over the three-year period was associated with a decreased intake of whole-grain products (FDR-*p* = 0.04). The decrease in UE was associated with a reduced intake of chocolate (FDR-*p* = 0.02), sweet pastries (FDR-*p* = 0.02), and savory pastries (FDR-*p* = 0.01). Furthermore, the decrease in EE was associated with increased intake of vegetables, fruit, and berries as a food group (FDR-*p* = 0.01) and separately with increased intake of vegetables (FDR-*p* = 0.02), fruit (FDR-*p* = 0.02), fatty creams (FDR-*p* = 0.02) and decreased intake of fatty cheeses (FDR-*p* = 0.049). Baseline BMI did not modify these associations, except for the association between the change in CR and the change in whole-grain products, which was no longer significant (FDR-*p* = 0.08).

**Table 2:** Linear regression analysis with changes in eating behavior traits (TFEQ-R18) and changes in food intake in the T2D-GENE intervention study (*n* = 368)

Outcome Variable	Predictor	Adjusted *R*^2^	b	SE	Standardized β	t	*p*-value	Adjusted *p*-value ^a^
**Food Groups** ^b^								
Δ Whole-grain Products		0.01						
	Δ CR		− 0.51	0.24	− 0.11	− 2.15	0.03*	0.04*
Δ Vegetables, Fruit and Berries		0.03						
	Δ EE		− 2.25	0.73	− 0.16	− 3.07	0.002**	0.01*
**Foods Within the Food Groups** ^b^								
Δ Sweet Pastries		0.02						
	Δ UE		0.46	0.19	0.13	2.45	0.02*	0.02*
Δ Fatty Savory Pastries		0.03						
	Δ UE		0.33	0.101	0.16	3.05	0.002**	0.01*
Δ Vegetables		0.02						
	Δ EE		− 0.90	0.34	-0.14	− 2.67	0.008**	0.02*
Δ Fruit		0.02						
	Δ EE		− 1.61	0.58	-0.14	− 2.77	0.006**	0.02*
Δ Fatty Cheeses		0.01						
	Δ EE		0.16	0.08	0.10	1.98	0.048*	0.049*
Δ Fatty Creams		0.02						
	Δ EE		− 0.11	0.05	-0.13	− 2.44	0.02*	0.02*
Δ Chocolate		0.02						
	Δ UE		0.14	0.05	0.15	2.96	0.003**	0.01*

Δ, delta value i.e. change from 0 to 3 years; CR, Cognitive Restraint; UE, Uncontrolled Eating; EE, Emotional Eating

^a^ Benjamini–Hochberg False Discovery Rate correction

^b^ Supplementary Material 2

FDR-*p* < 0.05*, FDR-*p* < 0.01**, FDR-*p* < 0.001***

## Discussion

As hypothesized, during the 3-year lifestyle intervention in aging prediabetic men, cognitive restraint (CR) scores increased, while uncontrolled eating (UE) and emotional eating (EE) scores decreased, even though the T2D-GENE intervention did not specifically target changes in eating behavior traits. Additionally, favorable changes were noted in the intake of various food groups, while the total energy intake did not change. Importantly, we found that changes in eating behavior traits were associated with dietary changes.

Our findings align with previous lifestyle interventions for individuals with or at risk for T2D, which have reported increased cognitive restraint and reduced overeating behaviors [31, 33, 34, 53], although they included smaller, mixed-sex samples and some even targeted eating behaviors [31, 53]. However, compared to other interventions utilizing the TFEQ-R18, the changes in eating behavior traits observed in our study were modest [53–55], possibly due to differences in study design, such as a specific emphasis on modifying eating behavior traits [53, 54], and population characteristics [53–55]. Our study involved a larger sample of older men, who typically exhibit higher CR scores and lower overeating tendencies, namely UE and EE, than younger individuals [25, 29, 56, 57], and baseline scores in our cohort reflected this pattern [53–55]. In our study, CR may have increased because of dietary counselling or an awareness of participating in an intervention. Future research should explore whether targeting eating behavior traits in interventions leads to greater changes in eating behavior traits in older men. 

As total energy intake remained unchanged, the observed shifts in food consumption suggest a qualitative improvement rather than only caloric restriction. The dietary changes were sustained throughout the 3-year period, indicating a stable shift in dietary behavior. Particularly the changes in the increased intake of fiber-rich foods and improved quality of dietary fat can be considered beneficial for people at risk of T2D, due to their positive effect on improving insulin sensitivity [52, 58]. Our findings among older men are in line with previous lifestyle interventions among middle-aged men and women with prediabetes or T2D [35, 36]. Changes in eating behavior traits seem to have a role in dietary changes among older men, regardless of BMI. In previous interventions, the increase in CR has been associated with improvement of the diet quality such as increased intake of fruit [31] and decreased energy intake [30] and fat, especially saturated fat [32] among men and women. Interestingly, the increase in CR was inversely associated with whole-grain product intake, despite an overall rise in whole-grain consumption during the intervention. This unexpected finding may reflect the nature of CR, which involves limiting food intake to manage body weight [19]. Although whole-grain products were encouraged, heightened CR may have led to a general reduction in food intake, including healthy options. As whole-grain bread is typically preferred by middle-aged and older Finnish men, and intake in our study aligned with population norms [14], the inverse association may indicate a broader decline in bread consumption. This reduction could be explained by more regular meal patterns or the replacement of bread-based snacks with fruits or vegetables, both emphasized during the intervention.

UE shares similarities with external eating (i.e. eating in response to the presence of food) as measured by the Dutch Eating Behavior Questionnaire [59]. Previously, a decrease in external eating was associated with decreased intakes of energy and fat among men and women [32] whereas in our study, the decrease in UE was associated with decreased intake of pastries and chocolate, – foods high in fat and energy. UE is derived from the original Three-Factor Eating Questionnaire traits disinhibition (i.e. lack of restraint of eating in response to negative feelings or the presence of palatable food) and susceptibility to hunger (i.e. susceptibility to feelings of hunger) [60]. Both UE and the related traits have been associated with higher intake of energy-dense foods in cross-sectional studies among men and women [23, 25, 28, 41, 49, 61], also supporting our findings. However, a longitudinal study observed that decreases of disinhibition and hunger were associated with increased intake of vegetables among men and women [31]. In conclusion, promoting the reduction in UE may benefit dietary improvements, and could help prevent T2D among older men. A reduction in UE may reflect improved regulation of reward-driven eating behavior. Given its association with reward sensitivity [62] and hedonic responses to palatable foods [63], lowered UE could indicate diminished reliance on highly palatable, nutrient-poor foods for pleasure, thereby enhancing dietary quality. While the absolute changes in intake associated with decreased UE were modest, they consistently targeted energy-dense foods high in saturated fat and sugar, suggesting a qualitative improvement in dietary choices that may have cumulative benefits for metabolic health over time.

EE has similarly been associated with increased consumption of hyperpalatable foods in cross-sectional studies [22, 25, 40–42, 61]. However, in our study, the decrease in EE was associated with an increased intake of vegetables, fruit, and berries and weakly with a reduced intake of fatty cheeses. This inverse relationship to the consumption of healthy foods, such as vegetables, is less commonly observed [64] making the results unexpected. Emotional eaters tend to regulate their emotions by excessive consumption of hyperpalatable foods, rich in fat and sugar, that provide an immediate reward and reduce the effect of stress [65]. The regular intake of hyperpalatable foods seems to gradually displace the consumption of health-promoting foods, such as vegetables and fruit [66], potentially explaining our findings. Our findings suggest that reducing EE may support better diet quality, but confirmation requires further research. To the best of our knowledge, no prior longitudinal studies have reported significant associations between changes in EE and diet.

Both UE and EE are associated with impulsive and emotion-driven eating, where food choices are often automatic or motivated by comfort-seeking [67, 68]. Thus, reductions in these traits may enable more conscious and deliberate food decisions, supporting healthier dietary patterns. We cannot confirm the effect of the intervention due to the absence of control group data, but during the additional meetings targeted at participants with higher BMI, topics such as mindful eating, flexible restraint, and identifying triggers for overeating, were emphasized. These strategies may have contributed to the observed changes by enhancing inhibitory control and promoting more intentional food choices. These findings support the potential of targeting impulsive eating traits in dietary interventions aimed at preventing T2D.

Our study adds to the limited body of research on eating behavior traits among aging men, an underrepresented population in this field. To our knowledge, no previous longitudinal studies have focused exclusively on men. As women typically score higher on measures of CR, UE, and EE compared to men [37–39] and given that the associations between eating behavior traits and dietary outcomes may differ by sex [21, 22], it is plausible that similar changes in eating behavior traits could have resulted in greater or qualitatively different dietary changes among women. Some cross-sectional studies have suggested that the associations between EE and sweet energy-dense foods may be stronger in women compared to men [23, 42], which could help explain the lack of such associations in our study. This may be attributed to sex differences in food preferences. A previous study indicated that EE is the main driver for snacking behavior at workplace, but among women it is more strongly reflected in the consumption of sweet energy-dense snacks [69]. Additionally, it has been found that women tend to prefer sweet comfort foods whereas men prefer more savory and meal-type foods, for example, pizza or pasta [70]. Furthermore, some cross-sectional studies have reported associations between EE and non-sweet energy-dense foods exclusively among men [25, 42]. Our results may provide some support for this, since the change in EE was directly, but weakly, associated with the change in the intake of fatty cheeses, which may be classified as non-sweet energy-dense foods, or at least, common ingredients in high-fat meals. Understanding the possible sex-specific aspects of eating behavior traits is crucial for more personalized prevention or treatment of non-communicable diseases such as T2D.

### Strengths and limitations

Our participants were aging prediabetic men with overweight or obesity, and thus the results might not be generalizable to all. However, individuals similar to our participants are those who need new effective ways to prevent T2D. Aging men are rare in an eating behavior study, and thus the results are novel. The number of participants stayed high although the food records and/or the TFEQ-R18 (from baseline and at three years) were not returned by all participants. Thus, this paper may lack statistical power to detect all possible changes and relationships. The absence of TFEQ-R18 and dietary data in the control group limits causal inference and prevents definitive attribution of observed changes to the intervention. However, this does not affect the validity of the observed associations between changes in eating behavior traits and dietary intake within the intervention group. These associations provide insight into how psychological factors may relate to dietary changes and help identify potential targets for future interventions.

The participants were likely more motivated to make dietary changes than the general population, as they were volunteers recently being made aware of their prediabetic status. Consequently, similar changes may be more difficult to achieve and maintain in less health-conscious or more diverse populations. This should be considered when interpreting the generalizability of the findings.

Previous studies on the associations between eating behavior traits and diet are mainly cross-sectional. We used a 3-year longitudinal analysis, providing more insight into causality than cross-sectional studies. In studies with a higher number of participants, food frequency questionnaires are more commonly used to assess the habitual food intake. We used 4-day food records that provide more accurate information of the actual food intake. Food records that include a weekend day and are kept for at least three days are commonly considered to represent the usual food intake [71]. Furthermore, the strength of food records is that they do not rely on participants’ memory. However, the amounts of foods might not be perfectly accurate because they are reported by the participants. We tried to increase accuracy with clear instructions before filling out the food records and checking them upon return with the participant. Another limitation is that the participants may change their usual eating habits or underreport due to the recording. However, this is more common among women and people with obesity [72–74].

Different eating behavior questionnaires are not entirely comparable even though they intend to assess the same phenomena. TFEQ-R18 has been validated among many populations [19, 22, 48] and it was developed to make it quicker and easier to measure eating behaviors [19]. Longer questionnaires, such as the original TFEQ with 51 items, might be too extensive for the participants to read or answer all questions accurately [60].

## Conclusion

Our unique study population provides novel results regarding longitudinal associations between eating behavior traits and diet in men. Our results suggest that in older prediabetic men, it might be favorable for food intake to support the decrease in two overeating tendencies, UE and EE, regardless of the BMI. The decrease in these two traits plays a role in the increased intake of vegetables, fruit, and berries and in decreased intake of certain energy-dense foods. These dietary changes could be beneficial for preventing T2D and other non-communicable diseases. Thus, the findings are important to consider when creating new prevention and treatment strategies. Further studies are needed to validate the causal relationship between both UE and EE and diet and to study the best methods to target UE and EE during an intervention.

## Supplementary Information

Below is the link to the electronic supplementary material.

Supplementary Material 1

Supplementary Material 2

Supplementary Material 3

Supplementary Material 4

## Data Availability

Due to ethical reasons, the data are not available. The lead author has full access to the data reported in the manuscript.

## **Abbreviations**

BMI: Body mass index (kg/m^2^)
CR: Cognitive restraint
EE: Emotional eating
FR: Food record
HbA1c: Hemoglobin A1c
TFEQ-R18: The three-factor eating questionnaire revised 18-item
T2D: Type 2 diabetes
UE: Uncontrolled eating
