# Supplementary material for: Changes in eating behavior traits and diet in older prediabetic men during a 3-year lifestyle intervention
Source: Eur J Nutr. 2026 Mar 5;65(3):80. doi: 10.1007/s00394-025-03876-7 (PMC12963192; doi:10.1007/s00394-025-03876-7)
Supplement: Supplementary file 1 — Supplementary Material 1 [file 394_2025_3876_MOESM1_ESM.docx]

Online resource 1 to: **Changes in Eating Behavior Traits and Diet in Older Prediabetic Men During a 3-year Lifestyle Intervention Conducted in Finland**

European Journal of Nutrition

Noora Koivu^a^ *, Maria Lankinen^a^,Ursula Schwab^a,b^

^a^ Institute of Public Health and Clinical Nutrition, University of Eastern Finland, P.O. Box 1627, FI-70211 Kuopio, Finland

^b^ Department of Medicine, Endocrinology and Clinical Nutrition, Kuopio University Hospital, Wellbeing Services County of North Savo, Kuopio, Finland

*Corresponding author: Noora Koivu, Institute of Public Health and Clinical Nutrition, Clinical Nutrition, University of Eastern Finland, P.O. Box 1627, FI-70211 Kuopio, Finland. Email address: [noora.koivu@uef.fi](mailto:noora.koivu@uef.fi)

**Supplementary Text 1** Detailed Description of the Three-Factor Eating Questionnaire (TFEQ-R18)

The Three-Factor Eating Questionnaire Revised 18-item (TFEQ-R18) [1] was used to assess the intensity of three eating behavior traits: (1) Cognitive restraint, CR (2) Uncontrolled eating, UE and (3) Emotional eating, EE. TFEQ-R18 is a shortened, revised version of the original 51-item TFEQ [2]. TFEQ-R18 contains 18 questions: 6 items assessing CR (items 2, 11, 12, 15, 16, 18), 9 assessing UE (items 1, 4, 5, 7, 8, 9, 13, 14, 17) and 3 assessing EE (items 3, 6, 10). Respondents provide answers on a 4-point Likert-scale, yielding scores between 1 and 4 for each item, with one item rated on an 8-point Likert scale. To ensure compatibility, scores are rescaled to 0–100 range for each eating behavior trait using the following formula: [(raw score – minimum raw score)/possible raw score range x 100] [3]. Higher scores indicate greater intensity of a specific eating behavior trait. In this study, a validated Finnish translation of the questionnaire was used; the original English version is included for reference and transparency.

*The Three-Factor Eating Questionnaire—Revised 18-Item*

1. When I smell a sizzling steak or juicy piece of meat, I find it very difficult to keep from eating, even if I have just finished a meal.

*Definitely true (4)/ mostly true (3)/ mostly false (2)/ definitely false (1)*

2. I deliberately take small helpings as a means of controlling my weight.

*Definitely true (4)/ mostly true (3)/ mostly false (2)/ definitely false (1)*

3. When I feel anxious, I find myself eating.

*Definitely true (4)/ mostly true (3)/ mostly false (2)/ definitely false (1)*

4. Sometimes when I start eating, I just can't seem to stop.

*Definitely true (4)/ mostly true (3)/ mostly false (2)/ definitely false (1)*

5. Being with someone who is eating often makes me hungry enough to eat also.

*Definitely true (4)/ mostly true (3)/ mostly false (2)/ definitely false (1)*

6. When I feel blue, I often overeat.

*Definitely true (4)/ mostly true (3)/ mostly false (2)/ definitely false (1)*

7. When I see a real delicacy, I often get so hungry that I have to eat right away.

*Definitely true (4)/ mostly true (3)/ mostly false (2)/ definitely false (1)*

8. I get so hungry that my stomach often seems like a bottomless pit.

*Definitely true (4)/ mostly true (3)/ mostly false (2)/ definitely false (1)*

9. I am always hungry so it is hard for me to stop eating before I finish the food on my plate.

*Definitely true (4)/ mostly true (3)/ mostly false (2)/ definitely false (1)*

10. When I feel lonely, I console myself by eating.

*Definitely true (4)/ mostly true (3)/ mostly false (2)/ definitely false (1)*

11. I consciously hold back at meals in order not to weight gain.

*Definitely true (4)/ mostly true (3)/ mostly false (2)/ definitely false (1)*

12. I do not eat some foods because they make me fat.

*Definitely true (4)/ mostly true (3)/ mostly false (2)/ definitely false (1)*

13. I am always hungry enough to eat at any time.

*Definitely true (4)/ mostly true (3)/ mostly false (2)/ definitely false (1)*

14. How often do you feel hungry?

*Only at meal times (1)/ sometimes between meals (2)/ often between meals (3)/ almost always (4)*

15. How frequently do you avoid “stocking up” on tempting foods?

*Almost never (1)/ seldom (2)/ usually (3)/ almost always (4)*

16. How likely are you to consciously eat less than you want?

*Unlikely (1)/ slightly likely (2)/ moderately likely (3)/ very likely (4)*

17. Do you go on eating binges though you are not hungry?

*Never (1)/ rarely (2)/ sometimes (3)/ at least once a week (4)*

18. On a scale of 1 to 8, where 1 means no restraint in eating (eating whatever you want, whenever you want it) and 8 means total restraint (constantly limiting food intake and never “giving in”), what number would you give yourself?

**References**

1. Karlsson J, Persson LO, Sjöström L, Sullivan M (2000). Psychometric properties and factor structure of the Three-Factor Eating Questionnaire (TFEQ) in obese men and women. Results from the Swedish Obese Subjects (SOS) study. *Int J Obes*. 24(12):1715-1725. doi:10.1038/sj.ijo.0801442
2. Stunkard AJ, Messick S (1985). The three-factor eating questionnaire to measure dietary restraint, disinhibition and hunger. *J Psychosom Res*. 29(1):71-83. doi:10.1016/0022-3999(85)90010-8
3. de Lauzon, B. de, Romon, M., Deschamps, V., Lafay, L., Borys, J. M., Karlsson, J., Ducimetiere, P., & Charles, M. A. (2004). Three-Factor Eating Questionnaire-R18 is able to distinguish among different eating patterns in a general population. *The Journal of Nutrition*, *134*(9), 2372–2380. https://doi.org/10.1093/jn/134.9.2372
