# Supplementary material for: Changes in eating behavior traits and diet in older prediabetic men during a 3-year lifestyle intervention
Source: Eur J Nutr. 2026 Mar 5;65(3):80. doi: 10.1007/s00394-025-03876-7 (PMC12963192; doi:10.1007/s00394-025-03876-7)
Supplement: Supplementary file 2 — Supplementary Material 2 [file 394_2025_3876_MOESM2_ESM.docx]

Online resource 2 for: **Changes in Eating Behavior Traits and Diet in Older Prediabetic Men During a 3-year Lifestyle Intervention Conducted in Finland**

European Journal of Nutrition

Noora Koivu^a^ *, Maria Lankinen^a^ Ursula Schwab^a b^

^a^ Institute of Public Health and Clinical Nutrition, University of Eastern Finland, P.O. Box 1627, FI-70211 Kuopio, Finland

^b^ Department of Medicine, Endocrinology and Clinical Nutrition, Kuopio University Hospital, Wellbeing Services County of North Savo, Kuopio, Finland

*Corresponding author: Noora Koivu, Institute of Public Health and Clinical Nutrition, Clinical Nutrition, University of Eastern Finland, P.O. Box 1627, FI-70211 Kuopio, Finland. Email address: [noora.koivu@uef.fi](mailto:noora.koivu@uef.fi)

**Supplementary Table 1** Categorization of food items from 4-day food records in T2D-GENE intervention

| **Food Group** | **Foods Included in the Food Group** |
| --- | --- |
| Low-fat Dairy | low-fat fermented dairy products (fat <1%), low-fat cheese (fat ≤17%), skimmed milk, vegetable oil cheeses, vegetable oil-based creams (fat <15%), low-fat creams (fat <10%) |
| Fatty Dairy | normal-fat milk (fat 1–1.5%), fatty milk (fat >1.5%), fatty fermented dairy products (fat >1 %), fatty cheeses (fat >17%), fatty creams(fat >10%) |
| Vegetables, Fruits and Berries | root vegetables, vegetables, legumes, mushrooms, fruit, berries |
| Whole-grain Products | whole-grain breads (fiber ≥ 6%), crispbreads (fiber ≥ 10%), whole-grain snack bars (fiber ≥ 6%), whole-grain flour and other whole-grain products (fiber ≥ 6%), whole-grain porridges, whole-grain cereals (fiber ≥ 6%) |
| Butter | butter and butter-based spreads |
| Non-tropical Vegetable Oil Products | non-tropical vegetable oils, vegetable oil-based spreads, liquid margarines |
| Fish | fresh, cooked and smoked fish |
| Non-sweet Energy-dense Foods | salty fatty pastries, mayonnaise, sausages, salty snacks (e.g. salted peanuts, chips, popcorn), fast food, fatty meat |
| Sweet Energy-dense Foods | sweet pastries, jams, ice-cream, chocolate, sweets, sugar, sherbets and popsicles, sugar-sweetened beverages, desserts |
| Alcoholic Beverages | mild alcoholic beverages, liquor, liqueur, wine |
| Nuts and Seeds | unsalted nuts, almonds, seeds and peanut butter |
