# Supplementary material for: Changes in eating behavior traits and diet in older prediabetic men during a 3-year lifestyle intervention
Source: Eur J Nutr. 2026 Mar 5;65(3):80. doi: 10.1007/s00394-025-03876-7 (PMC12963192; doi:10.1007/s00394-025-03876-7)
Supplement: Supplementary file 3 — Supplementary Material 3 [file 394_2025_3876_MOESM3_ESM.docx]

Online resource 3 for: **Changes in Eating Behavior Traits and Diet in Older Prediabetic Men During a 3-year Lifestyle Intervention Conducted in Finland**

European Journal of Nutrition

Noora Koivu^a^ *, Maria Lankinen^a^, Ursula Schwab^a b^

^a^ Institute of Public Health and Clinical Nutrition, University of Eastern Finland, P.O. Box 1627, FI-70211 Kuopio, Finland

^b^ Department of Medicine, Endocrinology and Clinical Nutrition, Kuopio University Hospital, Wellbeing Services County of North Savo, Kuopio, Finland

*Corresponding author: Noora Koivu, Institute of Public Health and Clinical Nutrition, Clinical Nutrition, University of Eastern Finland, P.O. Box 1627, FI-70211 Kuopio, Finland. Email address: [noora.koivu@uef.fi](mailto:noora.koivu@uef.fi)

**Supplementary Table 2** Pearson correlation coefficients between changes in eating behavior traits and changes in food intake in the T2D-GENE intervention study (n=368).

|  | **Δ Eating Behavior Traits** |  |  |
| --- | --- | --- | --- |
| **Δ Food Groups ^a^** |  |  |  |
|  | Δ CR | Δ UE | Δ EE |
| Δ Whole-grain Products | **-0.11*** | -0.07 | -0.03 |
| Δ Vegetables, Fruit and Berries | 0.06 | -0.08 | **-0.16**** |
| **Δ Foods Within the Food Groups ^a^** |  |  |  |
| Δ Sweet Pastries | -0.001 | **0.13*** | 0.003 |
| Δ Fatty Savory Pastries | -0.06 | **0.16**** | 0.07 |
| Δ Vegetables | 0.08 | -0.10 | **-0.14**** |
| Δ Fruit | 0.05 | -0.06 | **-0.14**** |
| Δ Fatty Cheeses | -0.07 | -0.03 | **0.10*** |
| Δ Fatty Creams | 0.04 | -0.04 | **-0.13*** |
| Δ Chocolate | -0.06 | **0.15**** | 0.03 |

Δ, delta value i.e. change from 0 to 3 years; CR, Cognitive restraint; UE, Uncontrolled eating; EE, Emotional eating

^a^  Food groups are presented in Supplementary table 1 (Online resource 2)

p<0.05*; p<0.01**
