# Supplementary material for: Changes in eating behavior traits and diet in older prediabetic men during a 3-year lifestyle intervention
Source: Eur J Nutr. 2026 Mar 5;65(3):80. doi: 10.1007/s00394-025-03876-7 (PMC12963192; doi:10.1007/s00394-025-03876-7)
Supplement: Supplementary file 4 — Supplementary Material 4 [file 394_2025_3876_MOESM4_ESM.docx]

Online resource 4 for: **Changes in Eating Behavior Traits and Diet in Older Prediabetic Men During a 3-year Lifestyle Intervention Conducted in Finland**

European Journal of Nutrition

Noora Koivu^a^ *, Maria Lankinen^a^ Ursula Schwab^a b^

^a^ Institute of Public Health and Clinical Nutrition, University of Eastern Finland, P.O. Box 1627, FI-70211 Kuopio, Finland

^b^ Department of Medicine, Endocrinology and Clinical Nutrition, Kuopio University Hospital, Wellbeing Services County of North Savo, Kuopio, Finland

*Corresponding author: Noora Koivu, Institute of Public Health and Clinical Nutrition, Clinical Nutrition, University of Eastern Finland, P.O. Box 1627, FI-70211 Kuopio, Finland. Email address: [noora.koivu@uef.fi](mailto:noora.koivu@uef.fi)

**Supplementary Table 3** Estimated Dietary Changes Over 3-Year T2D-GENE Intervention (n=368)

| **Food Group** | **Change from 0 to 3 years (g/day)** |
| --- | --- |
| Low-Fat Dairy Products | +33.6 |
| Fatty Dairy Products | -57.4 |
| Non-tropical Vegetable Oil Products | +12.8 |
| Butter | -4.8 |
| Wholegrain Products | +15.6 |
| Vegetables, Fruit and Berries | +78 |
| Non-sweet Energy-dense Foods | -13.1 |
| Sweet Energy-dense Foods | -29.4 |
| Alcoholic Beverages | -59.4 |
| Nuts and Seeds | +5.9 |
| Fish | +13.7 |
| **Foods Whitin Food Groups** |  |
| Fatty Creams | -1.5 |
| Vegetables | +28 |
| Fruit | +29.2 |
| Fatty Cheeses | -8.1 |
| Chocolate | -0.8 |
| Fatty Savory Pastries | -4.5 |
| Sweet Pastries | -4.8 |
